# Surface Chemistry of WC Powder Electrocatalysts Probed In Situ with NAP‐XPS

**DOI:** 10.1002/anie.202500965

**Published:** 2025-03-31

**Authors:** Christoph Griesser, Sergio Diaz‐Coello, Matteo Olgiati, Wanderson Ferraz do Valle, Toni Moser, Andrea Auer, Elena Pastor, Markus Valtiner, Julia Kunze Liebhäuser

**Affiliations:** ^1^ Department of Physical Chemistry University of Innsbruck Innrain 52 c Innsbruck 6020 Austria; ^2^ Institute of Applied Physics Vienna University of Technology Vienna 1040 Austria; ^3^ Instituto de Materiales y Nanotecnologia Departamento de Química Universidad de La Laguna Av. Astrofísico Francisco Sánchez s/n La Laguna Santa Cruz de Tenerife 38200 Spain

**Keywords:** Carbide, Electrocatalysis, Hydrogen evolution reaction, ICP‐MS, In situ XPS

## Abstract

Tungsten carbide (WC) is a renowned compound catalyst material for electrochemical water splitting, and its high electrocatalytic activity toward the hydrogen evolution reaction (HER) has been repeatedly reported. However, its susceptibility to oxidation raises the fundamental question of the underlying reason for its high activity, especially since passivation and thus potential deactivation can occur not only in air but also during reaction. Hence, the investigation of the surface chemistry under true operating conditions is crucial for a fundamental understanding of the electrocatalytic process. In this work, we use electrochemical X‐ray photoelectron spectroscopy (EC‐XPS) to revisit the surface chemistry of WC powder electrodes in alkaline electrolyte in situ and under full potential control. Our results show that although the surface is initially covered with oxide, this passive film dissolves in the electrolyte under electrochemical reaction conditions. This clarifies the active surface termination during the HER and highlights the potential of laboratory‐based EC‐XPS to study applied energy conversion materials.

## Graphical abstract

Tungsten carbide (WC) is a highly active catalyst for the HER. The reasons for its activity are, however, unclear due to its delicate susceptibility to oxidation and thus passivation. Electrochemical near ambient pressure XPS reveals dissolution of the passive film into the electrolyte under electrochemical working conditions, which effectively enables the electrocatalyst's enduring activity.

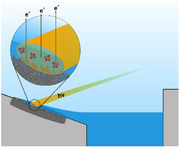

Over the past few decades, there has been substantial interest in transition metal (TM) compounds as potential alternatives to noble metals in electrocatalysis. This is mainly due to their structural and compositional diversity, which allows them to overcome the correlation of adsorption energies of intermediates, frequently referred to as scaling relations.^[^ ^1^ ^]^ In this context, TM carbides (TMCs) show promising potential, as highlighted by density functional theory (DFT) calculations, which reveal a break in scaling relations^[^ ^2^ ^]^ through their carbophobic and oxophilic characteristics as compared to the corresponding parent metals. Consequently, there has been a surge of interest in the application of TMCs as catalysts or support materials in (electro‐)catalytic processes, such as the hydrogen evolution reaction (HER),^[^ ^3^, ^4^, ^5^, ^6^, ^7^ ^]^ the CO_2_ reduction reaction (CO_2_RR),^[^ ^8^, ^9^, ^10^ ^]^ and the methanol and ethanol oxidation reactions, respectively.^[^ ^11^, ^12^ ^]^ Nevertheless, it is widely acknowledged that TMCs are susceptible to oxidation,^[^ ^13^, ^14^ ^]^ and such alterations in surface chemistry can significantly influence the theoretically proposed electrocatalytic performance. This tendency to oxidize is further complicated by the fact that oxidation may not only result from atmospheric exposure, which can typically be mitigated, but may also occur in situ, under electrochemical operating conditions.^[^ ^13^, ^14^ ^]^ The accurate experimental monitoring of these alterations under realistic conditions is extremely challenging, since the most direct method for studying interfacial chemistry—X‐ray photoelectron spectroscopy (XPS)—is not readily compatible with routine electrochemical experiments in aqueous solutions. However, the pioneering advances in near ambient pressure XPS (NAP‐XPS) technology^[^ ^15^, ^16^ ^]^ have enabled the direct combination of electrochemistry and XPS (EC‐XPS).^[^ ^17^ ^]^ The viability of EC‐XPS for in situ monitoring of electrode surface states has been demonstrated not only at various synchrotron facilities but also in laboratory settings.^[^ ^18^, ^19^, ^20^ ^]^ Consequently, EC‐XPS serves now as an ideal tool for tracking changes in surface chemistry under electrochemical operating conditions. We have recently^[^ ^18^ ^]^ developed an electrochemical setup for a lab‐based EC‐XPS system inspired by the work of Weingarth et al.,^[^ ^21^ ^]^ where the electrode is partially submerged in an electrolyte reservoir, leading to the formation of an ultrathin electrolyte film of less than 20 nm in thickness. This film is sufficiently thin to allow photoelectrons (PEs) emitted from the core levels of a given electrode material to escape from the solid/liquid interface, travel through the gas phase above the electrolyte, and enter the analyzer, while sufficient ionic conductivity in the in situ electrochemical experiment is ensured.

Utilizing this approach, we have revisited the surface chemistry of tungsten carbide (WC) in alkaline electrolyte. WC is an ideal model compound material and represents one of the most widely studied TMCs due to its platinum‐like properties^[^ ^22^ ^]^ proposed already in the 1970s by Levy and Boudart.^[^ ^23^ ^]^ This makes it theoretically very well‐suited for application in electrocatalysis, especially for the HER. It is, however, also known that oxide species reside at the WC surface.^[^ ^7^, ^24^, ^25^, ^26^ ^]^ These surface oxides can be partially removed through treatment with NaOH,^[^ ^24^ ^]^ but small residual amounts are expected to still persist at the surface and/or form in the electrolyte, potentially passivating and thereby deactivating it.^[^ ^27^, ^28^, ^29^ ^]^ Therefore, it is essential to monitor the true interface chemistry under electrochemical polarization conditions, i.e., during the electrochemical reaction of interest. Since alkaline electrolyzers are a highly relevant technology in the energy transition, knowledge of the electrocatalysts´ chemistry in alkaline electrolyte is most constructive toward technological progress. In this communication, we examine WC powder inks in terms of their electrochemical behavior during polarization in 0.1 M NaOH with particular focus on the surface chemistry under true operating conditions utilizing lab‐based EC‐XPS.

The layered SEM image (Figure 1a) shows that the WC powder has a particle size of about 200 nm. The overlaying energy dispersive X‐ray (EDX) maps (decomposed images are given in Figure S1) reveal that in addition to signals related to the presence of carbon (C K_α1,2_ edge) and tungsten (W M_α1_ edge), a significant contribution of oxygen (O K_α1_ edge) was found. This aligns well with ultra‐high vacuum (UHV) ex situ XPS experiments of the W 4f, O 1s, and C 1s regions (Figures 1b and S2A,B) and the survey spectrum (Figure S2C), showing that the pristine surface is primarily composed of WC, which is evident through the carbidic carbon signal at 283 eV and the carbidic W signal at 32.0 and 34.2 eV. The surface is also covered with a thin layer of WO_3_, as indicated by the W^6+^ XPS‐signal at 36.0 and 38.2 eV. The presence of oxide under ambient conditions has been reported in several studies.^[^ ^7^, ^25^, ^26^, ^30^ ^]^ An oxide layer thickness of 0.8 nm was estimated using Strohmeiers's equation^[^ ^31^ ^]^ (Supplementary Note 1). Furthermore, the absence of any additional signals in the survey spectrum (Figure S2C) proves that the ink is free of impurities. The absence of signals corresponding to tungsten oxides in the XRD patterns (Figure S3) indicates that only the surface has undergone oxidation, while the bulk of the powder has maintained its predominantly carbidic crystalline nature. Intriguingly, the powder ink prepared from this WC powder is still highly active toward the HER, as demonstrated by its electrochemical activity leading to the production of hydrogen as measured in situ with differential electrochemical mass spectrometry (DEMS) (see Figure 1c). The electrocatalytic activities of Pt and WO_3_ powder ink references are depicted in the same plot (for details of the electrode preparation procedures, see SI, Experimental Methods). The oxidized WC compound shows a remarkable electrocatalytic HER activity, evidenced by an onset overpotential of 0.1 V, which is close to the HER onset of Pt and significantly lower than that of WO_3_ at ∼ 0.5 V. The good electrocatalytic performance toward the HER is consistent with that reported in the existing literature.^[^ ^28^, ^30^, ^32^ ^]^ The knowledge of the surface chemistry, i.e., the presence of WO_3_ at the WC surface, however, requires further investigation of the system because this surface termination and the high HER activity seem to be clearly contradictory.

**Figure 1:**
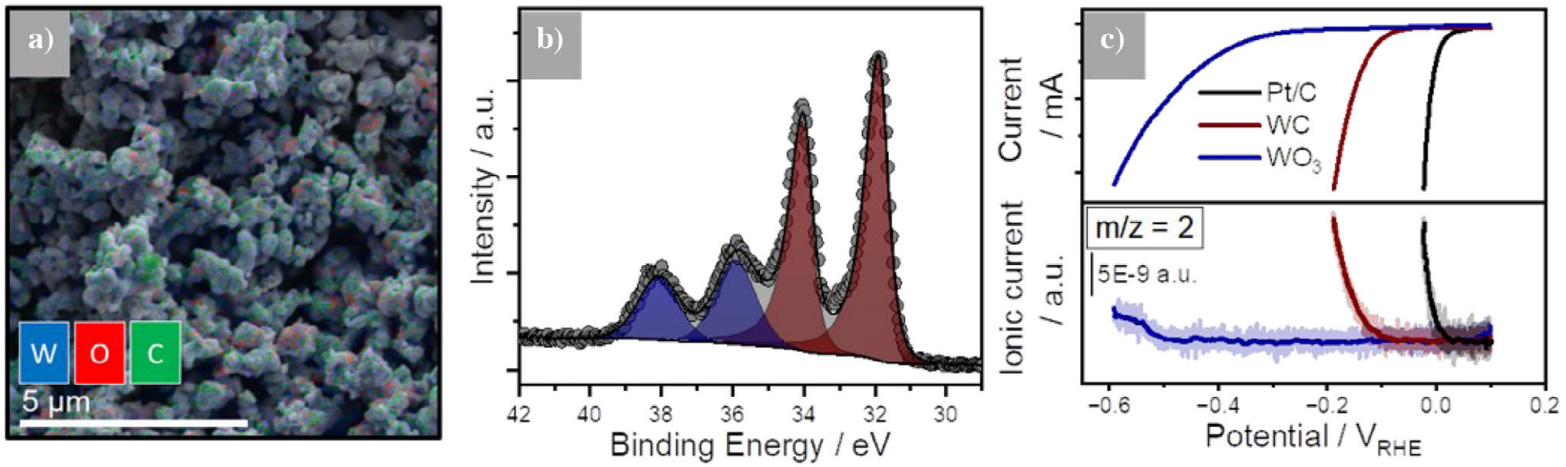
Morphology, chemistry, and electrocatalytic performance of the bare WC compound material. a) Layered scanning electron microscopy (SEM) image and energy dispersive X‐ray (EDX) maps of the WC powder, showing a particle size of ∼200 nm. b) UHV XPS high‐resolution spectrum of the W 4f region with significant WO_3_ contribution. c) Faradaic and ionic currents from differential electrochemical mass spectrometry (DEMS) of WC, WO_3_, and Pt/C powder inks in 0.1 M NaOH (scan rate 2 mV s^−1^).

To accurately address the question of why the WO_3_‐covered WC shows a high HER activity, even though bulk WO_3_ itself does not, in situ EC‐XPS experiments have been performed to gain insight into the surface chemistry under true electrochemical operating conditions. The EC‐XPS cell shown in Figure 2a and published earlier^[^ ^18^, ^33^ ^]^ was adapted to facilitate the investigation of powder ink electrodes with a glassy carbon (GC) current collector, in the present case. For a detailed description of the setup and its functionality, we refer to our previous work^[^ ^18^, ^33^ ^]^ and to an additional description of the adapted version used in this work in the Supporting Information (see Figure S4). All experiments were carried out at a temperature between 2°–8 °C leading to an H_2_O vapor pressure of ∼7–10 mbar, and under near ambient pressure conditions (∼10 mbar H_2_O back pressure). To gain a better understanding of the complex role of oxide formation and dissolution in WC powder ink catalysts, anodic polarization was deliberately performed prior to applying the HER conditions. This approach differs from standard investigations, where the HER reaction is typically studied by sweeping solely in the cathodic region, starting just above the thermodynamic potential for hydrogen evolution. In our experiments, the potentials were applied in the following sequence: 0.2 V_RHE_ (double‐layer region), 0.6 V_RHE_, 1.0 V_RHE_ (oxidation regime; see CV in Figure 2b), 0.2 V_RHE_ (double‐layer region), and −0.2 V_RHE_ (HER conditions). After polarization at a specific potential for 60 s, O 1s and W 4f regions were recorded consecutively, while the applied potential was held constant and the irradiated area for the EC‐XPS measurement was kept the same.

**Figure 2:**
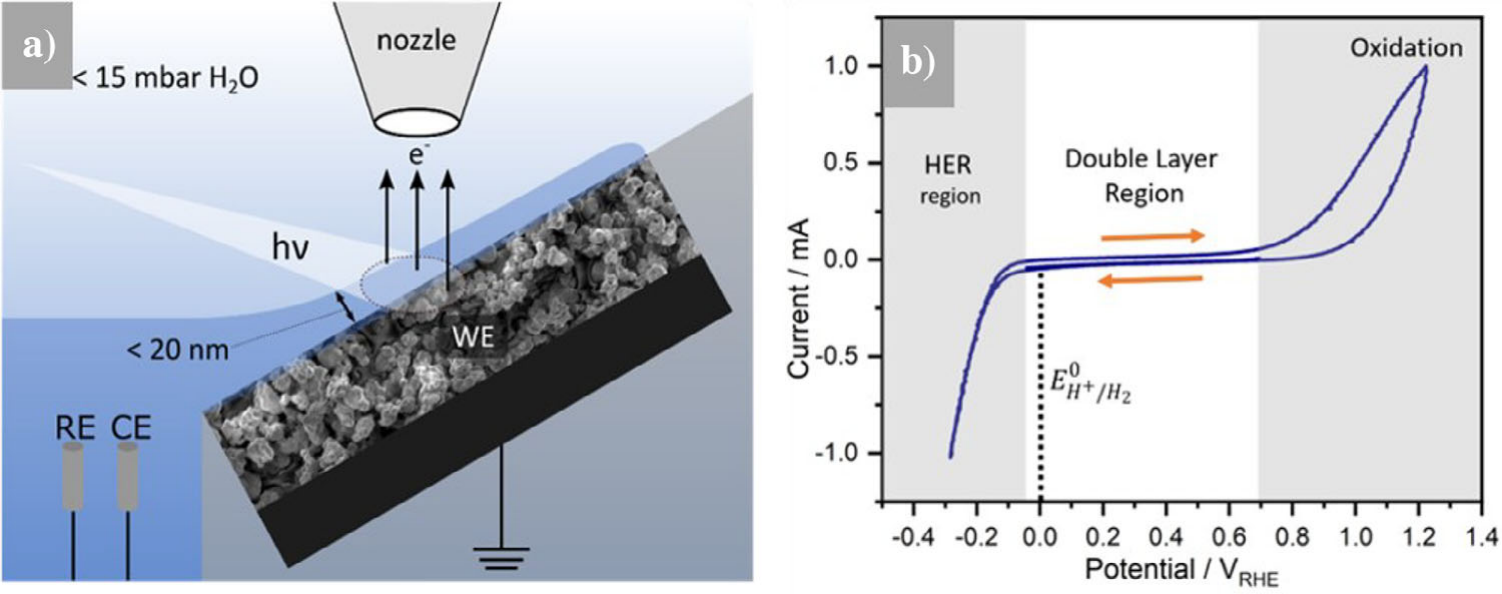
a) EC‐XPS in situ cell for powder materials. EC‐XPS PEEK cell with indent for the powder ink working electrode (WE), grounded via its glassy carbon (GC) current collector, platinum wires as quasi reference and counter electrodes (RE, CE). A tilted geometry allows for a < 20 nm thick 0.1 M NaOH electrolyte film connected to the bulk electrolyte reservoir. The measurement principle relies on cooling of the electrolyte to 2°–8 °C, leading to a vapor pressure of ∼7–10 mbar H_2_O and backfilling of the chamber with ∼10 mbar H_2_O. Details of the cell with the corresponding 3D drawing are given in the Supporting Information. b) Cyclic voltammogram (CV) of a WC powder ink in 0.1 M NaOH (scan rate: 50 mV s^−1^). Grey shaded regions indicate the potentials at which charge transfer takes place. Arrows indicate scan direction. The dotted line shows the thermodynamic potential of the HER.

At 0.2 V_RHE_, a potential at which no Faradaic current is visible in the CV, two distinct peaks can be observed in the O 1s region (Figure 3a). The signal at the lower binding energy (BE) edge of the spectrum (534.4 eV) is associated with liquid phase water, i.e., the electrolyte (referred to as LPW), while the signal at the higher BE (536.2 eV) corresponds to gas phase water (denoted as GPW). This split of the O 1s region is well known and described in the literature.^[^ ^17^, ^18^, ^34^ ^]^ The W 4f signal (Figure 3b) consists of two components. The doublet located at 31.9 and 34.1 eV perfectly matches the BE of WC and is in line with the UHV XPS measurement (see Figure 1b). The second component at 36.5 and 38.7 eV is assigned to a W^6+^ species, but its doublet is, however, significantly shifted by 0.5 eV compared to the WO_3_ doublet peak (at 36 and 38.2 eV) in the UHV XP spectrum (Figure 1b). A significant increase in the oxide‐to‐carbide ratio from 32:68 (pristine sample) to 50:50 (sample polarized at 0.2 V_RHE_) is observed. The peak positions described above are based on careful peak fitting; details regarding the fitting procedure are given in Supplementary Note 2.

**Figure 3:**
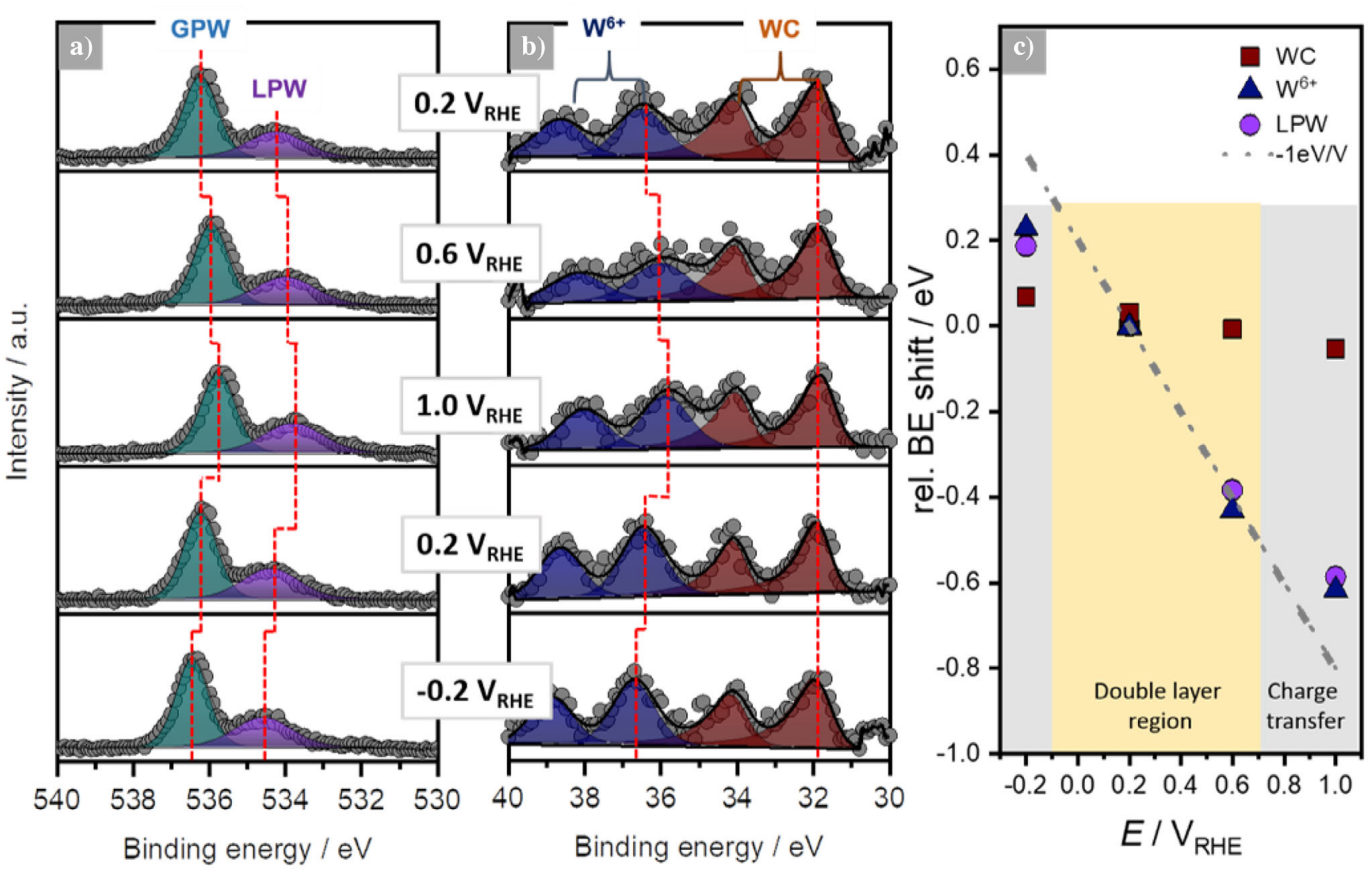
EC‐XP spectra and peak shifts of the WC powder ink electrode in 0.1 M NaOH. a) O 1s high‐resolution spectra with liquid phase water (LPW) and gas phase water (GPW) signals, showing distinct shifts in binding energy (BE) with altered potential. b) W 4f high‐esolution spectra with WC and W^6+^ components, where WC shows negligible shift in BE with potential, while the W^6+^ peak positions significantly change. In a) and b), the spectral responses to the first applied potential are shown in the top panels; the potential changes are carried out from top to bottom. c) Quantitative evaluation of the BE shifts of all components calculated corresponding to Equation (1); the dashed line indicates the expected −1eV V^−1^ shift in the double layer region, where no charge transfer reactions occur. The grey shaded areas highlight the potential regions in which charge transfer reactions take place.

To determine the spectro‐electrochemical properties of this interface, the potential of the WE was consecutively and systematically altered (the topmost panels in Figure 3a,b show the spectral responses to the first applied potentials; the order of potential changes is from top to bottom). The electrochemical BE shift of the LPW component in the O 1s spectra (Figure 3a,c) is determined as −1 eV V^−1^ between 0.2 V_RHE_ (534.4 eV) and 0.6 V_RHE_ (534.0 eV), with deviations from the unity slope observed at potentials beyond the nominal double layer region of the system, i.e., at < −0.2 V_RHE_ and > 1.0 V_RHE_. This indicates the influence of charge transfer contributions (compare Supplementary Note 3 for further information). The GPW component (Figure S5) shifts by −0.75 eV V^−1^ in the apparent double layer region and also deviates from unity slope at −0.2 V_RHE_ and 1.0 V_RHE_. The lower shift in BE of the GPW is expected as the vacuum level of the GPW couples to the vacuum level of the electrolyte surface and to the vacuum level of the analyzer nozzle.^[^ ^34^ ^]^ The electrochemical shifts of species inside the ion‐conducting electrolyte are characteristically observed in in situ EC‐XPS experiments; see, e.g., Refs. [17, 18, 34] and can be explained by the grounding of the WE alongside the analyzer. Due to this grounding, the complete potential drop, i.e., the potential difference between the polarized solid electrode and the electrolyte, is reflected through the electrochemical BE shift of the LPW peak, which primarily originates from the bulk electrolyte (see Figure S6). This provides evidence that the electrolyte film in the probed area (300 µm spot size) is conductively connected to the electrolyte reservoir and that the performed spectro‐electrochemical experiments are thus meaningful. The stability of the experiment is demonstrated by stepping the potential back to 0.2 V_RHE_ during the potential step experiment; the second spectrum measured at 0.2 V_RHE_ is identical in peak position and only deviates slightly in full width at half maximum (FWHM) values (see Figure 3 and Table S1). Moreover, no significant changes in the integrated peak areas of the LPW and GPW signals (Table S2) are observed, which confirms a stable and consistent electrolyte film thickness throughout the experiment.

In the W 4f region, altering the potential from 0.2 V_RHE_ to 0.6 V_RHE_ and 1.0 V_RHE_ results in a significant change of the spectral shape as the two previously well distinguishable doublets begin to overlay. Deconvolution of the spectra reveals that the WC doublet does not shift with altered potential, which is expected due to the alignment of the Fermi levels of the electrode and the analyzer.^[^ ^17^, ^18^, ^34^ ^]^ However, the WO_3_ signals shift from 36.5 eV (W 4f_7/2_) and 38.7 eV (W 4f_5/2_) at 0.2 V_RHE_ to 36.0 eV and 38.2 eV at 0.6 V_RHE_ and to even lower BE values at 1.0 V_RHE_. To further interpret the EC‐XPS data, the changes in BE of all the relevant components have been calculated relative to the BEs at 0.2 V_RHE_, where no specific electrochemical reaction is expected, and are plotted in Figure 3c:

(1) $${\mathrm{relBE}}_{i}^{\mathrm{shift}}=\mathrm{BE}{\left(E\right)}_{i}-\mathrm{BE}{\left(E=0.2V_{\mathrm{RHE}}\right)}_{i}$$

from this it is evident that the W^6+^ signal (Figure 3c, blue triangles) exhibits a shift that closely mirrors the shift of the LPW feature (Figure 3c, violet circles), not only in the double layer region (−1 eV V^−1^ from 0.2 V_RHE_ to 0.6 V_RHE_) but also by showing the same deviation from the unity slope at 1.0 V_RHE_ and −0.2 V_RHE_, i.e., at potentials where significant current is observed. This strongly indicates that the oxide is a chemical species that is not electronically connected to the WE and that is therefore not grounded alongside the analyzer. Instead, this suggests that the oxide is dissolved in the electrolyte, which makes the oxide species experience the same interfacial potential difference, i.e., electrochemical shift, as the bulk electrolyte.

Although at first glance counter‐intuitive, a detachment of WO_3_ from the WE could also explain the increase in the oxide‐to‐carbide ratio after immersion of the electrode in the electrolyte solution. During the EC‐XPS experiment, the WE is covered with an ultrathin electrolyte layer, which attenuates the PEs ejected from the core levels of the WE, and thereby diminishes the intensity of all related signals. However, if it is assumed that the oxide dissolves as WO_4_^2−^ in the electrolyte, the PEs ejected from WO_4_^2−^ are less attenuated by the electrolyte. Consequently, the signal from the WO_4_^2−^ (denoted as W^6+^ in Figure 3b) is more intense as compared to the UHV XPS experiment, which leads to an increase in the oxide‐to‐carbide ratio.

Expectedly, the EC‐XPS results perfectly agree with the experimental Pourbaix diagram of WC measured by Weidman et al.,^[^ ^35^ ^]^ which suggests surface oxidation/dissolution at potentials more positive than 0.5 V_RHE_. Furthermore, the Pourbaix diagram of W in aqueous solution indicates that the oxide is not stable in alkaline electrolyte and dissolves as WO_4_^2−^.^[^ ^36^ ^]^ Therefore, traces of WO_4_^2−^ should be detectable in the electrolyte. To verify this, inductively coupled plasma mass spectrometry (ICP‐MS) coupled with a flow cell setup^[^ ^37^ ^]^ was employed (details are provided in the Experimental Methods section, and a sketch of the flow cell is given in Figure S7), which allows for the detection of trace amounts of W, independent of its chemical state, under reaction conditions. In this experiment (Figure 4), the WE was subjected to several potential sweeps, where a clear signal related to dissolved W was detected at anodic potentials, with an onset at ∼0.75 V_RHE_, where surface oxidation and dissolution of the oxide take place.

**Figure 4:**
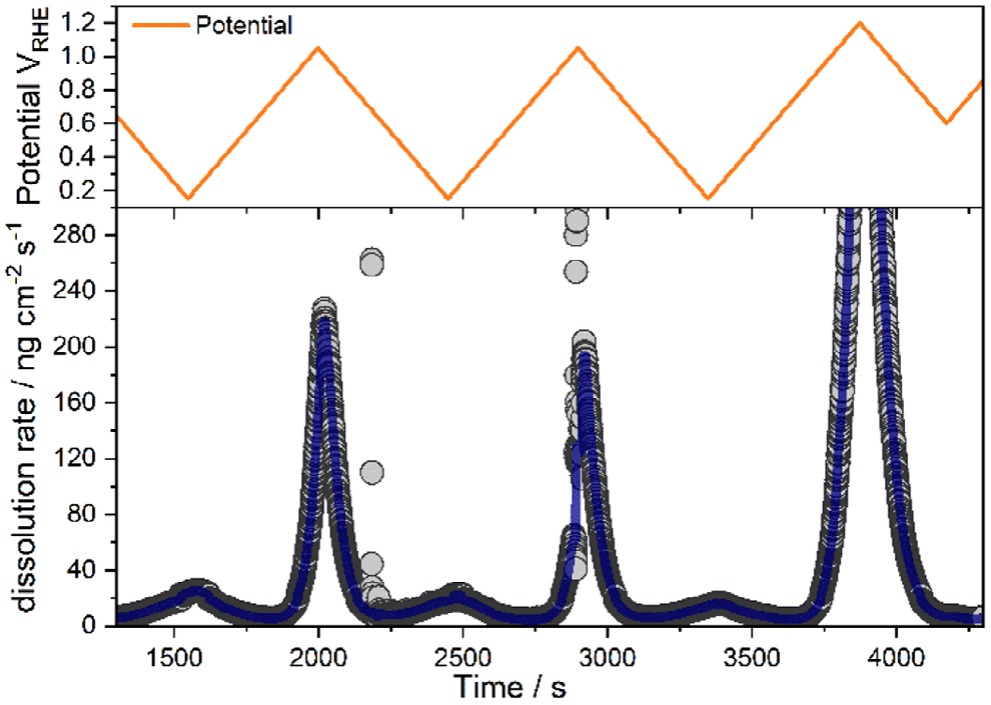
Online ICP‐MS analysis of the WC powder ink electrode in 0.1 M NaOH. W dissolution rate (bottom panel) at the corresponding potentials (top panel) versus time during potential sweeps (three cycles) with a scan rate of 2 mV s^−1^. The upper vertex potential was increased from 1.0 V_RHE_ (first two cycles) to 1.2 V_RHE_ (third cycle).

The formation of a stable oxide film passivating the surface can be excluded because the dissolution rate stays constant over several cycles and increases with increasing anodic potential. If passivation occurred, a significant decrease in dissolution rates over cycles would be expected.^[^ ^27^ ^]^ To exclude the effect of steady potential changes in the sweeping experiments, potential steps (Figure S8) were applied to the WC electrode, where the same behavior, i.e., a strong correlation between potential and dissolution rate, was found. During the potential sweep experiment (Figure 4), small peaks appear in all three cathodic scans when the potential reaches a value of ∼ 0.45 V_RHE_. The same behavior is observed in negative going potential steps, e.g., from 0.5 V_RHE_ to 0.3 V_RHE_ (see Figure S8). A possible reason for this is a coulostatic repulsion of the negatively charged WO_4_^2−^ ions during the negative potential pulse, i.e., the related increase of negative charge at the WE. This is also in agreement with the slight increase in intensity of the W^6+^ signal after stepping from 1.0 V_RHE_ to 0.2 V_RHE_ (compare Figure 3 and Table S2) in the EC‐XPS experiment. Overall, the online ICP‐MS experiments are well in line with the EC‐XPS data. To further validate both the EC‐XPS approach and the ICP‐MS results, ex‐situ emersion XPS and EDX experiments (experimental details are given in the Supporting Information) were conducted after polarization at specific potentials (1.0 V_RHE_, 0.4 V_RHE_, i.e., open circuit potential [OCP], 0.2 V_RHE_, −0.2 V_RHE_, and −0.6 V_RHE_). No significant variations were observed in the XPS (Figure S9) and/or the EDX spectra (Figure S10) across the different applied potentials. The absence of W^6+^ signals indicates the effective dissolution of surface WO_3_ as WO_4_^2−^ after emersion and rinsing. It is noteworthy that in the W 4f region (Figure S9B), all spectra consistently display a slight shoulder at 35 eV, indicating the presence of small amounts of suboxides. These findings strengthen the hypothesis that in the potential region investigated in this paper, the oxide dissolves in the electrolyte. This can explain the high activity of the WC powder ink electrocatalyst toward the HER, and its clear superior performance compared to the WO_3_ reference (see Figure 1).

In summary, this work highlights the capability of EC‐XPS to study not only the electrode‐electrolyte interface of planar metal substrates, such as metal foils, but also that of more applied powder ink electrocatalysts. This is demonstrated through the in situ EC‐XPS investigation of WC as a model compound material. WC is typically covered by an ultrathin WO_3_ layer, as confirmed with UHV‐XPS, while the surface oxide is found to detach from the electrode surface upon exposure to 0.1 M NaOH as WO_4_^2−^, and a presumably oxide‐free surface is active as WE in the examined potential range (−0.2 V_RHE_ to 1.0 V_RHE_; compare Figure S11).

Oxide dissolution is also confirmed with ICP‐MS experiments. This behavior explains the high electrochemical activity toward the HER, as well as the strong correlation between theoretical predictions and experimental results. Our findings underscore the critical role of EC‐XPS in directly probing the interfacial chemistry, offering insights about the catalyst surface under real in situ conditions. This technique, besides the proof of concept presented in this work, can be extremely valuable in the study of beyond state‐of‐the‐art catalysts for applied electrochemical research and energy conversion.

## Supporting Information

The authors have cited additional references within the Supporting Information.^[^ ^38^, ^39^, ^40^ ^]^

## Conflict of Interests

The authors declare no conflict of interest.

## Supporting information



Supporting Information

## Data Availability

The data that support the findings of this study are openly available in InvenioRDM at https://doi.org/10.48323/n20mb‐16c53.
