# Supplementary material for: Surface Chemistry of WC Powder Electrocatalysts Probed In Situ with NAP‐XPS
Source: Angew Chem Int Ed Engl. 2025 Mar 31;64(26):e202500965. doi: 10.1002/anie.202500965 (PMC12184310; doi:10.1002/anie.202500965)
Supplement: Supplementary file 1 — Supporting Information [file ANIE-64-e202500965-s001.docx]

Supplementary Information for

**Surface Chemistry of WC powder electrocatalysts probed in situ with NAP‑XPS**

Christoph Griesser^a†^, Sergio Diaz-Coello^a†^, Matteo Olgiati^b^, Wanderson Ferraz do Valle^b^, Toni Moser^a^, Andrea Auer^a^, Elena Pastor^c^, Markus Valtiner^b^ and Julia Kunze‑Liebhäuser^a^

a) Department of Physical Chemistry, University of Innsbruck, Innrain 52c, Innsbruck Austria

b) Institute of Applied Physics, Vienna University of Technology, 1040 Vienna

c) Departamento de Química, Instituto de Materiales y Nanotecnologia, Universidad de La Laguna, PO Box 456, 38200, La Laguna, Santa Cruz de Tenerife, Spain

**This PDF file includes:**

Experimental methods

Supplementary figures

Supplementary tables

Supplementary notes

References

**Experimental methods**

**Physicochemical Characterization**

Scanning electron microscopy (SEM) images and energy dispersive X-ray spectroscopy maps (EDS-maps) of the catalyst were acquired using a TESCAN Clara microscope. The as-received WC powder was immobilized on conductive carbon adhesive tabs supported on an aluminum sample holder. The sample was introduced in the main chamber of the SEM instrument, and the surface was scanned with an electron beam energy of 10 keV. Secondary electron images were obtained using an Everhart–Thornley detector. Additionally, EDS-maps of the surface were recorded using an Ultim Max 65 EDS detector (Oxford Instruments). The resulting EDS spectra identified signals corresponding to tungsten, carbon, and oxygen.

The X-ray diffraction (XRD) measurement of the powder material has been conducted using a PANanalytical X’Pert Pro universal diffractometer with Cu Kα radiation (λ = 0.1550 nm) generated at 40 kV and 20 mA. The patterns were collected at a scan rate of 0.04° s^–1^ over a 2θ range of 20° to 85°. The crystalline phases of the diffraction pattern have been identified and assigned accordingly to the ICDD PDF-2 database.

Ultrahigh vacuum X-ray photoelectron spectroscopy (XPS) analysis of the catalyst powders was performed using a Thermo Scientific Multilab 2000 system, equipped with a hemispherical analyzer and a monochromated Al Kα X-ray source (1486.6 eV). Charge compensation was achieved using a flood gun. High-resolution spectra were acquired with a 650 μm spot size at a fixed angle of 0° relative to the surface normal, using 0.05 eV step size, 20 eV pass energy, and a dwell time of 0.1 s.

**Electrodes and ink preparation**

Catalyst inks were prepared from commercial WC powders (Alfa Aesar, 99.5%) and WO_3_ powders (Aldrich, 99.995%) by ultrasound-assisted formation of catalytic ink. Briefly, 20 mg of WC were mixed with 15 µL of Nafion (Sigma-Aldrich, 5 wt%) solution and 500 µl of isopropanol (Merck, p.a.) for 30 minutes. The ink was drop-casted onto different glassy carbon (GC) disks and dried under Ar (Messer, ≥ 99.995%) flow to achieve a final amount of 4 mg·cm^-2^ of catalyst supported on the surface. According to the necessities of each experiment, different GC disks with different diameters were used. In particular, standard electrochemistry experiments and online inductively coupled plasma mass spectrometry (ICP-MS) were recorded by drop-casting the catalytic ink onto a 7 mm disk (A_geo_ = 0.385 cm^2^), differential electrochemical mass spectrometry (DEMS) experiments were recorded on a 5 mm disk (A_geo_ = 0.196 cm^2^) and the in-situ electrochemical X-ray photoelectron spectroscopy (EC-XPS) experiments were recorded on a 10 mm disk (A_geo_ = 0.785 cm^2^). Prior to each drop-cast, the respective GC disk was polished by using a diamond dispersion (Struers, 1 µm) and then subsequently rinsed in ultrapure water (MilliQ, 18.2 MΩ·cm). Unless expressed otherwise, all the experiments have been done by using a flame-annealed graphite rod as an auxiliary electrode; and a Hg/HgO electrode (ALS-Japan) contained in a Luggin capillary filled with 0.1 M NaOH. All potential expressed in this work have been transformed and expressed versus the hydrogen reversible electrode (RHE).

**Differential electrochemical mass spectrometry**

The accurate onset overpotential of alkaline hydrogen evolution reaction has been solved by means of differential electrochemical mass spectrometry. In this case, the experiments were conducted in a PEEK-based three-electrode flow cell (Hidden, Type A) controlled by an Autolab PGSTAT204 potentiostat/galvanostat. The former was coupled to the entrance of a mass spectrometer (Hidden, HPR-40) using a hydrophobic PTFE membrane (Goretex). Then, the working electrode was placed closed to the latter to achieve a thin layer configuration of the supporting electrolyte, which was previously deaerated in an external PTFE reservoir for 20 minutes. Both WC and WO_3_ supported inks were immersed at 0.1 V_RHE_ and then, the potential was sweep towards cathodic potentials at 1 mV·s^-1^. The equipment was set to follow the mass-to-charge m/z = 2 as it is the sole fragment of molecular hydrogen ([H_2_]^+^). The ionization of the molecules has been achieved by means of an IrO_2_-based filament operating at 70.0 V while the detection of the ionized fragments has been caried out by using a secondary electron multiplier detector. Additional information about the determination of the delay time for hydrogen detection in this equipment can already be found elsewhere.[^⁠1^](#_CTVL001ccb06b47caf04c90a3be195f9717147d)

**Electrochemical X-ray photoelectron spectroscopy**

All electrochemical in-situ measurements were conducted at a commercial SPECS NAP-XPS system. The setup includes a monochromatic small-spot Al Kα X-ray source (μFocus 600 NAP, 300 μm spot size), a vertically oriented hemispherical energy analyzer (PHOBIAS 150 NAP), and a μ-metal main chamber for shielding against external electric fields. It also features a separately pumped preparation chamber and a 4-axis manipulator (x, y, z directions and polar rotation), equipped with electrical feed-throughs for potential control and thermocouples, as well as cooling pipes. The experiments were conducted in a home-made PTFE-based three-electrode half-cell whose potential was controlled by using an Autolab PGSTAT204 potentiostat/galvanostat. Further technical details, including a detailed description of the measurement process can be found in ref[^⁠2^](#_CTVL00109972d3d026c4e709be6b25ba223ee3c)[^,3^](#_CTVL00104afa2f5dd74488e848a82179d3c4e14). At this configuration, the potential was changed from 0.2 V_RHE_ to 1.0 V_RHE_ by applying steps for 400 mV. Afterwards, the potential was stepped back to 0.2 V_RHE_ and finally taken to – 0.2 V_RHE_. Thus, at every potential step, spectra were collected at a pass energy of 50 eV with an energy step size of 0.01 eV for the regions corresponding to oxygen (O 1s; 530 – 540 eV) and tungsten (W 4f; 30 to 40 eV). All experiments were carried out at a background water pressure of about 8 mbar and a temperature between 2-8 °C. In the same way as the classic XPS experiments, the results have been analyzed with the CASAXPS software by applying a Shirley-type background.

**Online inductively coupled plasma mass spectrometry**

Elemental dissolution during the potential swept was evaluated with Inductively Coupled Plasma Mass Spectrometry (ICP-MS, Agilent 7900 ICP-MS, Agilent Technologies), equipped with a collision cell that uses a flow of helium (5 mL min^-1^) as cell gas. Calibrations were performed prior to measurements by using a standard solution consisting of 58 ppm of Na_2_WO_4_·2H_2_O (Sigma Aldrich, > 99%) dissolved in ultrapure water.

The coupled electrochemical-ICP-MS experiments were performed using a home-built flow cell, which was already reported in former publications[^⁠4^](#_CTVL0016f08203c26ed4549b91665ded6dbe69f). A schematic drawing of the cross section of the flow cell is reported in Fig. S7. In short, the flow cell is made out of PEEK and PTFE and allows for electrochemistry in a three-electrodes configuration consisting of a leak-free Ag/AgCl reference electrode (RE), a Pt rod as counter electrode (CE) and WC-glassy carbon as working electrode (WE). Leakage of electrolyte was prevented by pressing the flow cell on the WE by means of a 3 mm diameter o-ring, thereby allowing for a total exposed area of approximately 0.07 cm^2^.

Flow of the analyte solution was controlled with pressurized Ar to achieve a flow rate of approximately 5.8 ± 0.4 mg of solution per second[^⁠4^](#_CTVL0016f08203c26ed4549b91665ded6dbe69f). Before being nebulised and introduced to the plasma torch, the analyte flowing out of the electrochemical flow cell was mixed with a standard solution, where Yttrium was used as internal standard. A delay time of ~16s was determined.

**Ex-situ emersion XPS and ex-situ emersion EDX**

Additional ex-situ emersion characterization of the material's surface and bulk chemistry was carried out with XPS and EDX. For this experiment, the WC powder ink was drop-casted onto glassy carbon current collectors. Potentials of either -0.6 V_RHE_, -0.2 V_RHE_, 0.2 V_RHE_, OCP (0.4 V_RHE_), or 1.0 V_RHE_ were applied for 1000 seconds using a three-electrode half-cell configuration. Afterwards, the powder ink electrodes were emersed from the cell, rinsed with ultrapure water (MilliQ, 18.2 MΩ·cm), dried under a stream of Ar, and placed on a sample holder to conduct UHV-XPS and EDX experiments.

XPS was carried out in a Multilab 2000 ESCA equipped with an Alpha 110 hemispherical sector analyzer (Thermo Fisher Scientific). Monochromatized Al Kα (1486.6 eV) X-rays were used. Charge compensation was provided by a flood gun (electron emission at 6 eV kinetic energy). Survey scans were recorded at a pass energy of 100 eV and an energy step size of 1 eV, and high-resolution spectra were recorded at a pass energy of 20 eV and an energy step size of 0.1 eV for the W 4f region. Charge-induced shifts of the signals were corrected with reference to the WC component (31.9 eV).

EDX studies were conducted with an electron beam energy of 10 keV. Secondary electron images were measured using an Everhart–Thornley detector. Additionally, EDX maps were recorded using an Ultim Max 65 detector (Oxford Instruments). The resulting spectra show signals corresponding to tungsten (W), carbon (C), and oxygen (O).


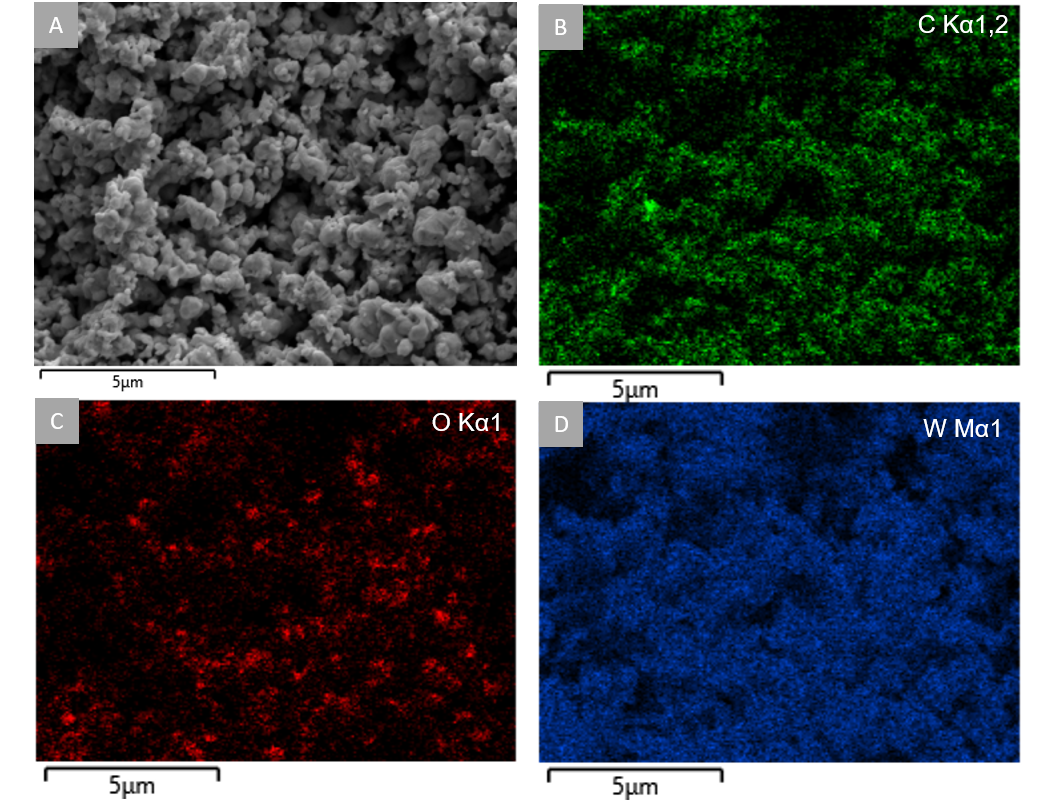


**Figure S1.** Scanning electron microscopy image (A) and energy dispersive X-ray maps of the carbon (B), oxygen (C) and tungsten (D) for the as-received WC powder material.


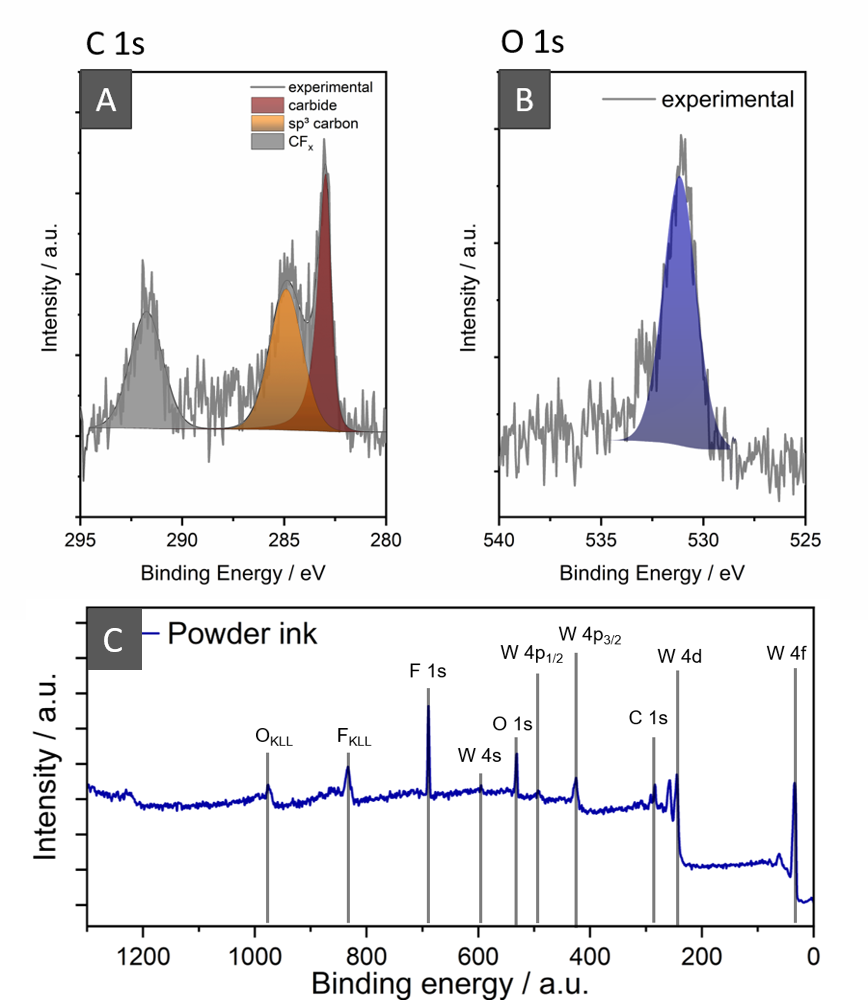


**Figure S2.** UHV-XP spectra of the C 1s (A) and O 1s (B) regions of the WC powder ink electrode. (A) A clear signal at about 283 eV highlights the carbidic nature of the WC electrode. Contributions from sp³ carbon and CF_x_ functionalities are visible due to the Nafion ionomer. (B) an increase of the O 1s region is observed which agrees well with the W 4f spectrum shown in the main paper (Fig. 1B), where also contributions of WO_3_ to the W4f region are visible. (C) only signals corresponding to the WC ink are observed, indicating that the ink is free of detectable impurities.

**Supplementary note 1. Oxide film thickness determination**

To estimate the thickness of the oxide films on top of the WC electrode an exponential decrease of signal intensity throughout the overlayer was assumed resulting in the following equation[^⁠5^](#_CTVL0015963444506b94f80932e586d01880205)*:*

$$d_{ox}=-\Lambda_{ox}*cos\Theta*ln\left( ( \frac{N_{m}\Lambda_{m}I_{ox}}{{N_{ox}\Lambda_{ox}I}_{m}})+1 \right)$$

$\Lambda_{ox}$/$\Lambda_{m}$ represent the IMFP of the photoelectron in the metal/oxide respectively and were determined utilizing the NIST Electron Inelastic-Mean-Free-Path Database (1.5 nm for WC 2.4 nm for WO_3_).$\Theta$ is the take off angle between the electrode and the analyzer (0°).

$I_{ox}$/$I_{m}$ represents the intensity of the W 4f signal related to the metal/oxide respectively. The fitted spectrum is given in Fig. 1B (main paper). *N_ox_* and *N_m_* are the atom densities of W and WO_3_. Regarding the atom density of W in WC and in WO_3_ we assumed a density of 15.63 g/cm³ for the carbide and of 7.16 g/cm³ for the oxide. We want to note here that this is a rough estimation on the oxide thickness. With that we found an oxide layer thickness of about 0.8 nm (this corresponds to about a double layer of oxide on top of the carbide).


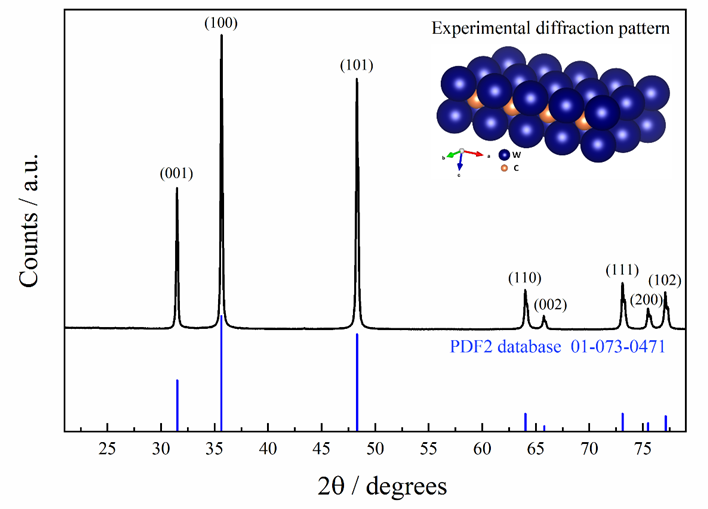


**Figure S3.** Diffraction pattern of the pristine WC powder. The experimental pattern is shown in black, while the corresponding peak assignments based on the 01-073-0471 PDF2 structure database are given in blue. The inset displays the model derived from the corresponding CIF file and visualized using VESTA software ^6^.


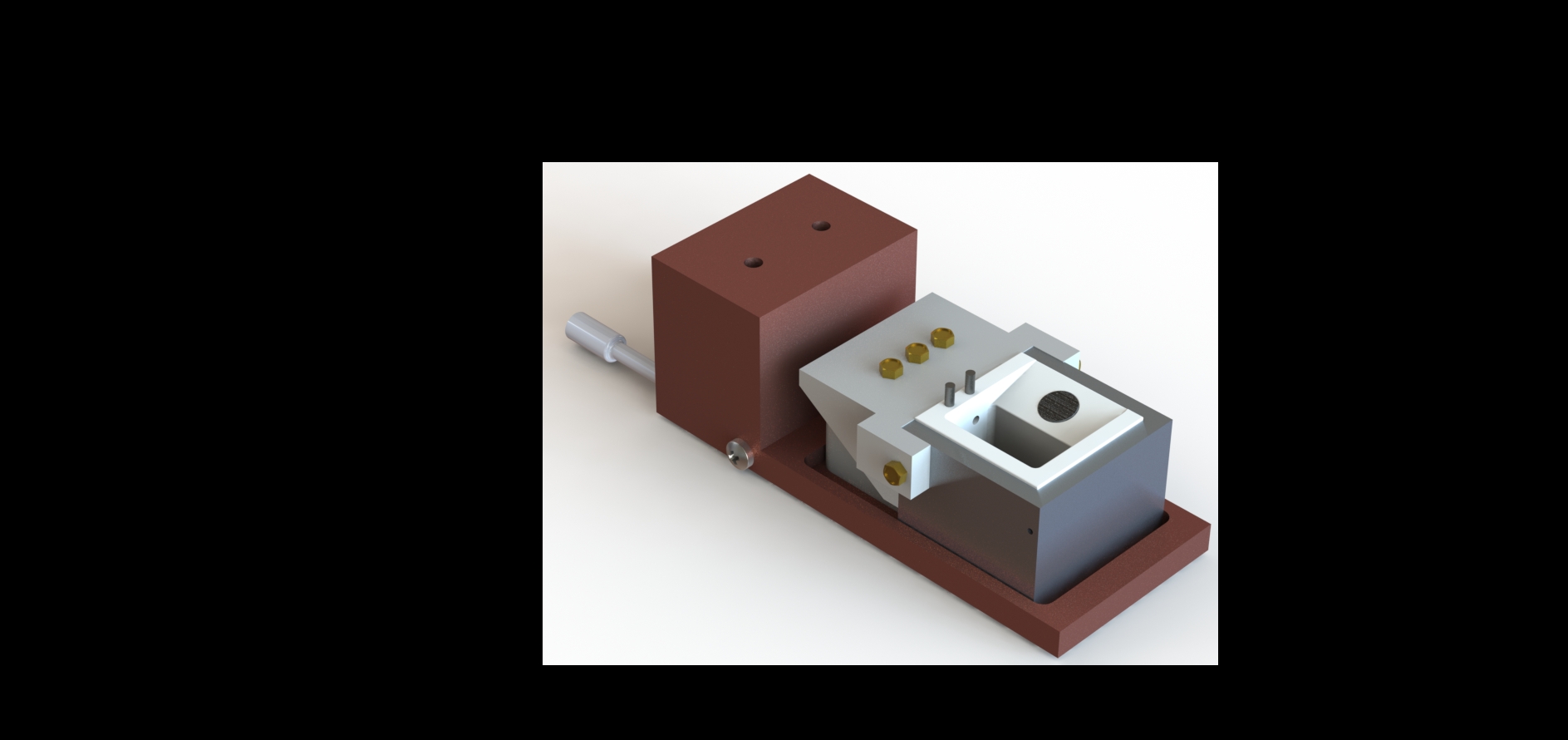


**Figure S4.** 3D sketch of the electrochemical setup used for the *in-situ* experiments. Details regarding the setup is given in refs.[^⁠2^](#_CTVL00109972d3d026c4e709be6b25ba223ee3c)[^,3^](#_CTVL00104afa2f5dd74488e848a82179d3c4e14). The cell is adapted so that a glassy carbon disk with a diameter of 10 mm fits into the indentation in the cell body. Hence any given powder ink can be drop-casted on the GC disk, which serves as current collector, and be investigated.

**Supplementary note 2. Peak fitting procedure**

XPS spectra were acquired with a pass energy of 50 eV in constant analyzer energy mode. Peak fitting was carried out using CasaXPS software [^⁠7^](#_CTVL001afcecf8927e745cf8856af744e606c3b), and no binding energy corrections were applied, so the spectra reflect the raw data presented in Data S1. This approach avoids complications from peak shifts induced by potential-dependent binding energies, particularly for electrolyte species, though this is not an issue for species directly connected to the electrode.

A Shirley-type background was used for all spectra. For the O 1s signals, mixed Gaussian-Lorentzian (GL30) functions were employed. In the W 4f spectra, the spin-orbit splitting was fixed at 2.18 eV (25), with an A(0.35, 0.64, 10)GL(10) function applied to the carbide signal and a mixed Gaussian-Lorentzian (GL30) function used for oxidized tungsten. The FWHM of the respective spin orbit couples was set equal. No further constraints were applied.

**Supplementary note 3. Electrochemical shift of the liquid phase water (LPW) peak**

In principle, the LPW peak should shift for -1eV/V (assuming a metal liquid interface) for an ideal polarizable electrode, however there are cases where a deviation is expected:

1. The electrolyte film is decoupled from the bulk electrolyte reservoir and hence from the reference and counter electrode. In this case there is no electrochemical shift expected and hence no proper EC-XPS can be conducted.
2. The double layer region extends far into the electrolyte so that it cannot be assumed that the LPW signal is only due to PEs ejected from water molecules which experience the full potential drop. In this case a deviation smaller than |-1eV|/V as well as an asymmetric peak shape would be expected. This could be the case in very diluted solutions or very thin electrolyte films. [^⁠8^](#_CTVL0016efd6678893b4ae0a122d250ff923f95)[^,9^](#_CTVL001ce1f697d0ec44d5d90c766f8767a22e8)
3. The film is poorly connected to the electrolyte reservoir. Hence a shift significantly smaller than |-1eV|/V is expected
4. Significant currents, caused e.g. by charge transfer reactions, lead to an iRs drop caused by the resistivity of the ultrathin nature of the ultrathin electrolyte film. Here a deviation smaller than |-1eV|/V is expected.

As in the double layer the slope perfectly matches -1eV/V only deviating at potentials where significant current is observed we conclude that case for causes the deviation from unity slope. The shift of the electrolyte peak on a semiconductor electrolyte interface is thoroughly discussed in ref [^⁠9^](#_CTVL001ce1f697d0ec44d5d90c766f8767a22e8).


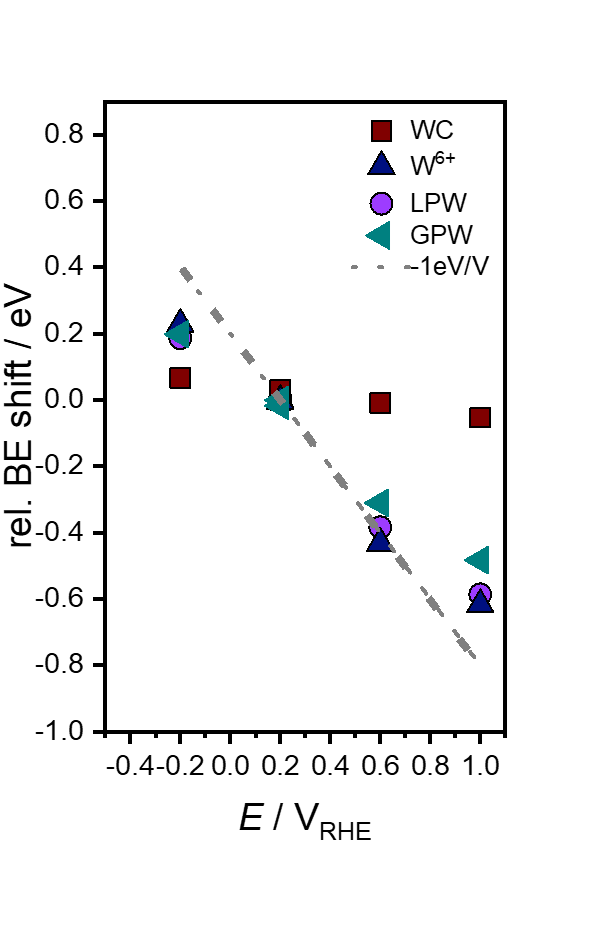


**Figure S5.** Relative binding energy shift of all species. Additional to the components shown in Fig. 3C also the relative BE shift of the GPW is depicted.


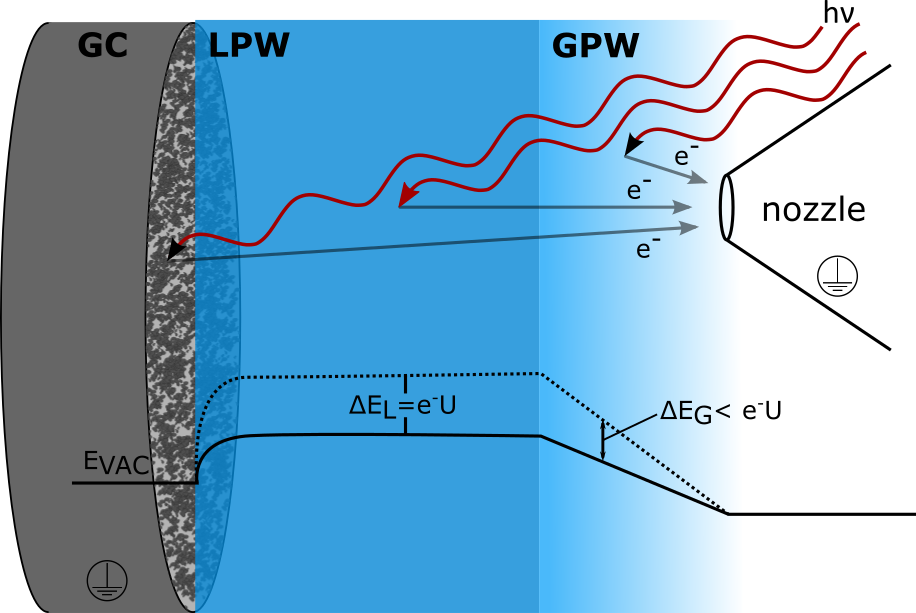


**Figure S6.** Scheme of the potential drop in the EC-XPS experiment. Due to the grounding of the sample alongside the analyzer no electrochemical shift is expected for any given species electrically connected to the WE. As the thickness of the electrochemical double layer is small compared to the overall film thickness a -1 eV/V shift is expected for species in the electrolyte (e.g. LPW), due to the interfacial potential drop. A smaller shift is expected for the GPW component as the GPW vacuum level is coupled to both the analyzer front cone and the water. For a detailed description of the behavior of the GPW we refer to Ref.[^⁠9^](#_CTVL001ce1f697d0ec44d5d90c766f8767a22e8).

**Table S1.** Peak positions and full width at half maximum (FWHM) of the components shown in Figure 1 and Figure 2 (main paper) used for the construction of Figure 2C (main paper).

| Potential / V_RHE_ | B.E. [FWHM] W 4f_7/2_ / eV | | B.E. [FWHM] O 1s / eV | |
| --- | --- | --- | --- | --- |
|  | WC | WO_x_^y-^ | LPW | GPW |
| Pristine* | 32.0 [0.7] | 36.0 [1.2] |  |  |
| 0.2 | 31.9 [1.0] | 36.5 [1.5] | 534.4 [2.0] | 536.2 [1.0] |
| 0.6 | 31.9 [1.0] | 36.0 [1.8] | 534.0 [1.7] | 535.9 [1.0] |
| 1.0 | 31.9 [0.9] | 35.9 [1.5] | 533.8 [1.8] | 535.8 [1.0] |
| 0.2 | 31.9 [0.8] | 36.5 [1.3] | 534.4 [1.9] | 536.2 [1.0] |
| -0.2 | 32.0 [1.0] | 36.7 [1.3] | 534.6 [1.9] | 536.4 [1.0] |
|  |  |  |  |  |
| Peak shape | A(0.35,0.64,10)GL(10) | GL(30) | GL(30) | GL(30) |
| Asymmetry parameter | 0.28 | 0.28 | 0.00 | 0.00 |
| BE constraint** | Arbitrary | Arbitrary | Arbitrary | Arbitrary |
| FWHM*** constraint | Arbitrary | Arbitrary | Arbitrary | Arbitrary |
|  |  |  |  |  |
| * This experiment was conducted in UHV at a Thermo Scientific Multilab 2000 system, with a pass energy of 20 eV, so the FWHMs are expected to be smaller compared to the in-situ experiments.  ** The splitting of the W4f signal was set to 2.18 eV.  *** The FWHM of the W 4_7/2_ and W4f _5/2_ was set to be equal. | | | | |

**Table S2.** Peak area portions of the O 1s and W 4f regions.

| Potential / V_RHE_ | Peak area portion of W 4f region/ % | | Peak area portion of O 1s region/ % | |
| --- | --- | --- | --- | --- |
|  | WC | WO_4_^2-^ | LPW | GPW |
| Pristine | 68 | 32 |  |  |
| 0.2 | 51 | 49 | 63 | 37 |
| 0.6 | 47 | 53 | 64 | 36 |
| 1.0 | 46 | 54 | 61 | 39 |
| 0.2 | 38 | 62 | 61 | 39 |
| -0.2 | 44 | 56 | 61 | 39 |

**
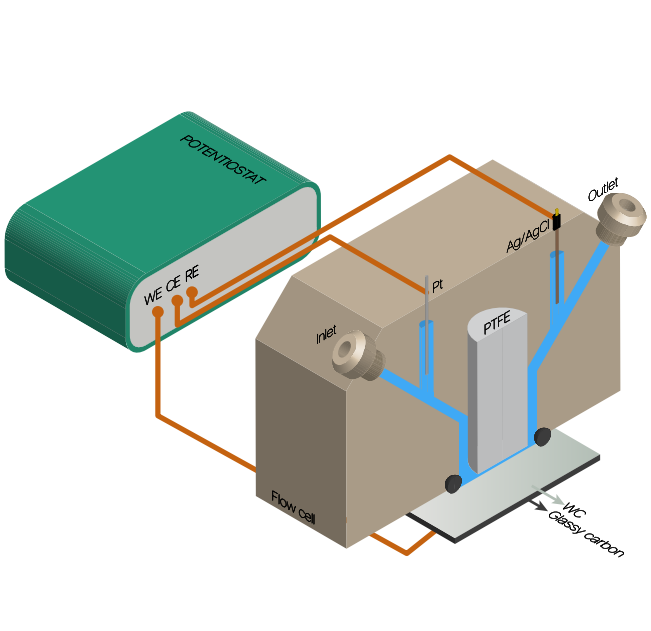
**

**Figure S7.** Sketch of the ICP-MS flow cell. The flow cell is made out of PEEK and PTFE and allows for electrochemistry in a three-electrodes configuration consisting of a leak-free Ag/AgCl reference electrode (RE), a Pt rod as counter electrode (CE) and WC-glassy carbon as working electrode (WE). Leakage of electrolyte was prevented by pressing the flow cell on the WE by means of a 3 mm diameter o-ring, thereby allowing for a total exposed area of approximately 0.07 cm^2^.

**
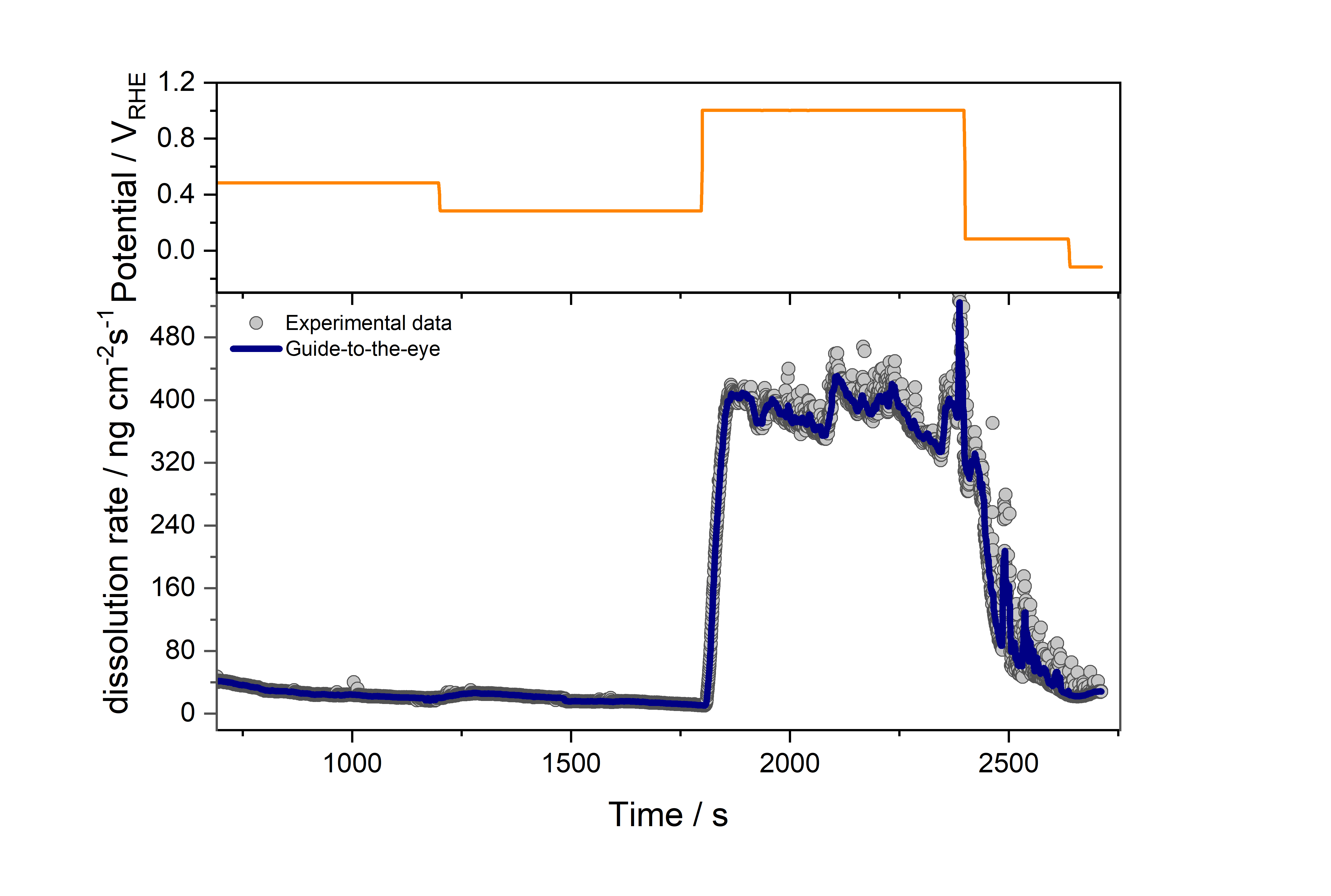
**

**Figure S8.** Online ICP.MS analysis of the WC powder ink electrode in 0.1 M NaOH. W dissolution (bottom panel) at the corresponding potential (top panel). Potentials were stepped from 0.5 V_RHE_ 🡪 0.3 V_RHE_ 🡪 1.0 V_RHE_ 🡪 0.0 V_RHE_ 🡪 -0.1 V_RHE_.


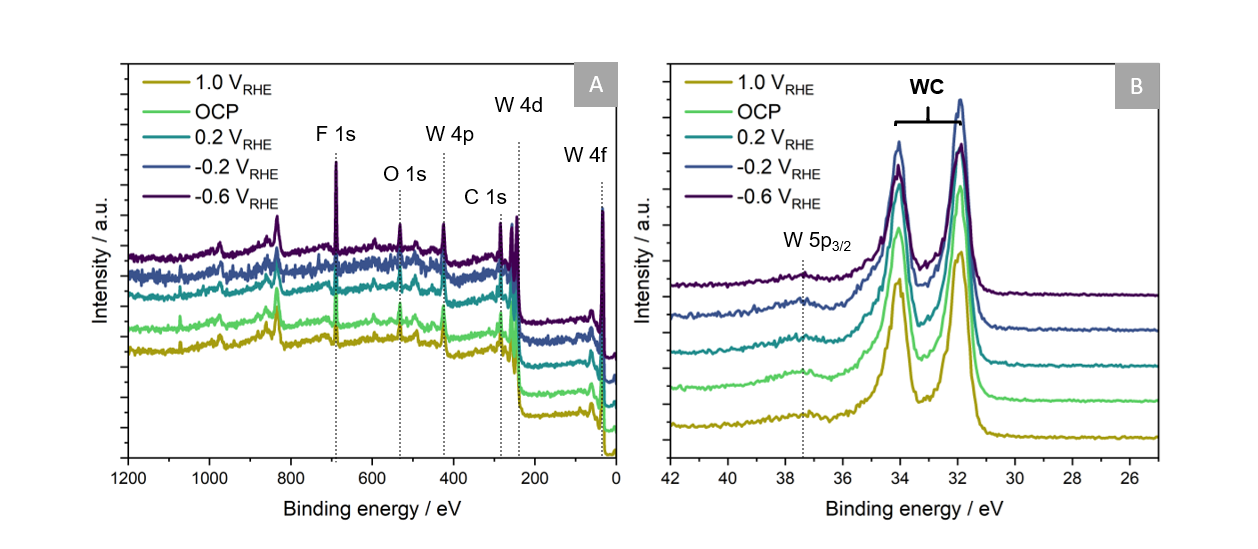


**Figure S9.** Ex-situ emersion XPS experiments of the WC powder ink electrode after polarization at different potentials. (A) survey spectra and (B) W 4f region with signals of WC at 31.9 and 34.1 eV. The shoulder at 35 eV in the W 4f region indicates the presence of small amounts of suboxides. The signal at 37.3 eV is attributed to the W 5p3/2 of the WC.

**
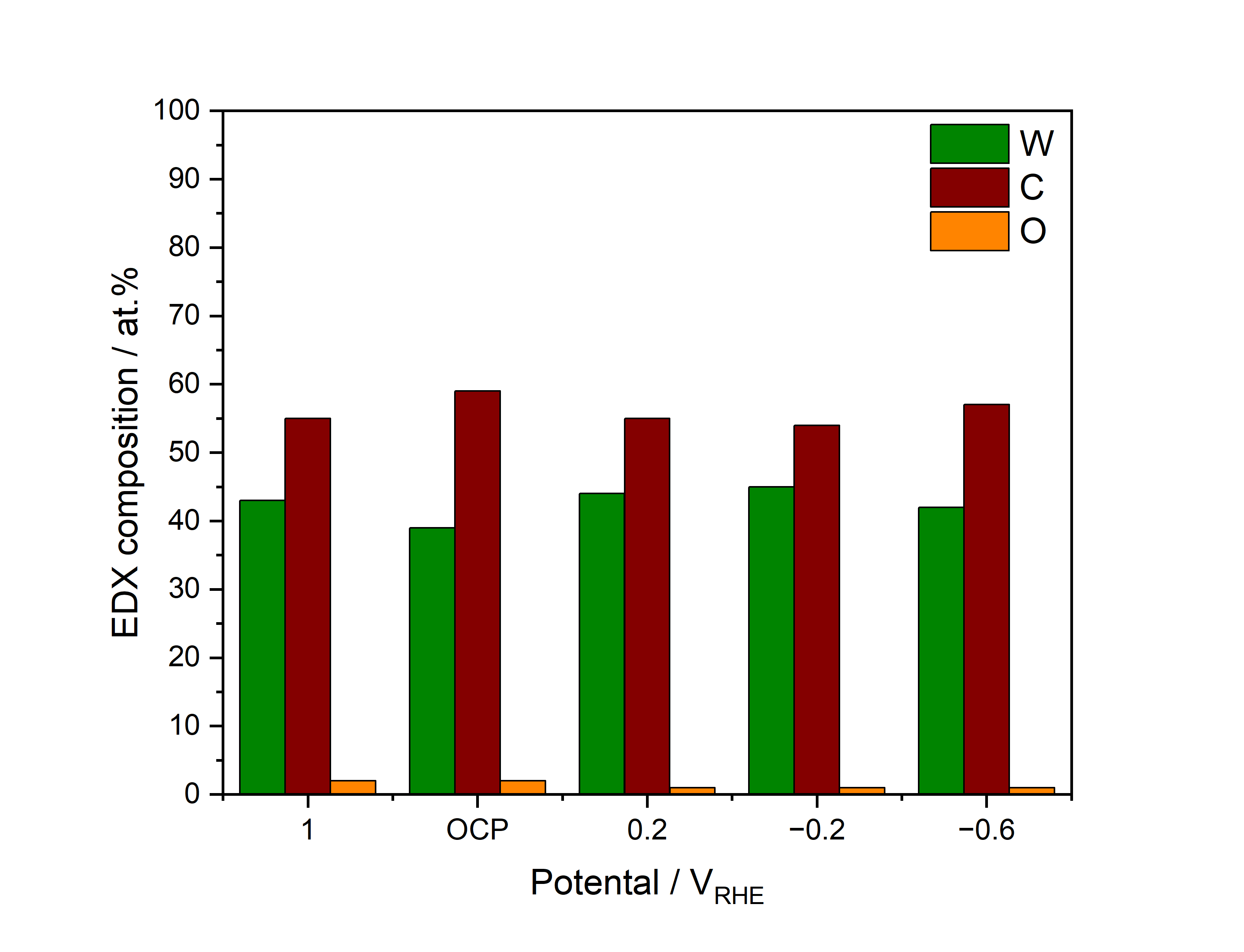
**

**Figure S10.** Quantification of ex-situ EDX experiments performed after polarization at different potentials for 15 minutes. The evaluation does not account for fluorine species, as this is related to the Nafion binder solely.


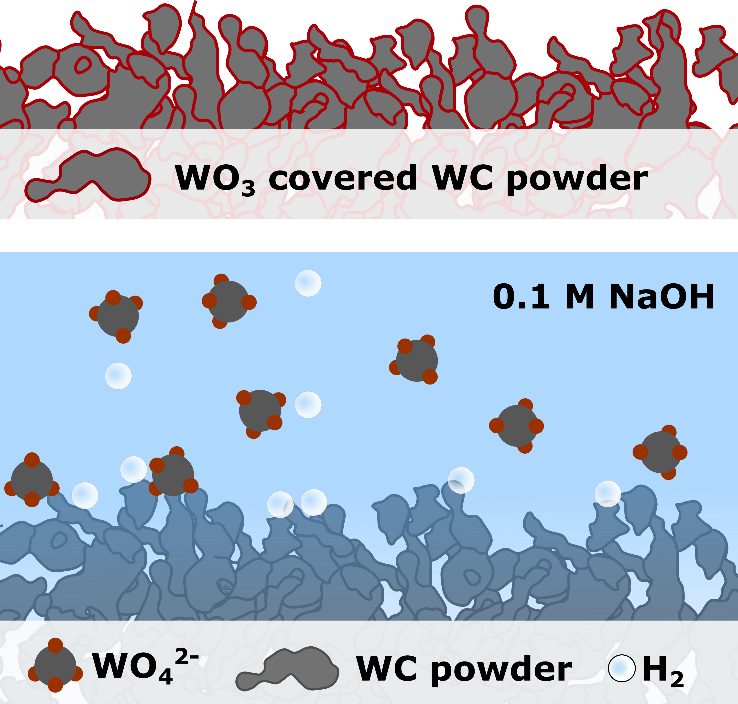


**Figure S11.** Scheme illustrating the surface chemistry change under electrochemical polarization conditions. In the top panel, the WC powder ink electrode is initially covered by an ultrathin WO_3_ film. After immersion and polarization in the electrolyte (bottom panel), the surface oxide dissolves as WO₄²⁻, leaving a predominantly bare WC surface that controls the surface chemistry during HER operation. Note that the relative sizes are not to scale.

REFERENCES

1. Díaz-Coello, S., Winkler, D., Griesser, C., Moser, T., Rodríguez, J. L., Kunze-Liebhäuser, J., García, G. & Pastor, E. Highly Active W2C-Based Composites for the HER in Alkaline Solution: the Role of Surface Oxide Species. *ACS applied materials & interfaces* **16,** 21877–21884; 10.1021/acsami.4c01612 (2024).

2. Griesser, C., Winkler, D., Moser, T., Haug, L., Thaler, M., Portenkirchner, E., Klötzer, B., Diaz‐Coello, S., Pastor, E. & KunzeLiebhäuser, J. Lab‐based electrochemical X‐ray photoelectron spectroscopy for in‐situ probing of redox processes at the electrified solid/liquid interface. *Electrochemical Science Advances*; 10.1002/elsa.202300007 (2023).

3. Haug, L., Griesser, C., Thurner, C. W., Winkler, D., Moser, T., Thaler, M., Bartl, P., Rainer, M., Portenkirchner, E., Schumacher, D., Dierschke, K., Köpfle, N., Penner, S., Beyer, M. K., Loerting, T., Kunze-Liebhäuser, J. & Klötzer, B. A laboratory-based multifunctional near ambient pressure X-ray photoelectron spectroscopy system for electrochemical, catalytic, and cryogenic studies. *The Review of scientific instruments* **94**; 10.1063/5.0151755 (2023).

4. Dworschak, D., Cheng, H.-W., Ku, C.-S., Chiang, C.-Y., Lin, C.-H. & Valtiner, M. Comparison of elemental resolved non-confined and restricted electrochemical degradation of nickel base alloys. *Corrosion Science* **190,** 109629; 10.1016/j.corsci.2021.109629 (2021).

5. Strohmeier, B. R. An ESCA method for determining the oxide thickness on aluminum alloys. *Surface & Interface Analysis* **15,** 51–56; 10.1002/sia.740150109 (1990).

6. Momma, K. & Izumi, F. *J Appl Crystallogr* **44,** 1272–1276; 10.1107/S0021889811038970 (2011).

7. Fairley, N., Fernandez, V., Richard‐Plouet, M., Guillot-Deudon, C., Walton, J., Smith, E., Flahaut, D., Greiner, M., Biesinger, M., Tougaard, S., Morgan, D. & Baltrusaitis, J. Systematic and collaborative approach to problem solving using X-ray photoelectron spectroscopy. *Applied Surface Science Advances* **5,** 100112; 10.1016/j.apsadv.2021.100112 (2021).

8. Favaro, M., Jeong, B., Ross, P. N., Yano, J., Hussain, Z., Liu, Z. & Crumlin, E. J. Unravelling the electrochemical double layer by direct probing of the solid/liquid interface. *Nature communications* **7,** 12695; 10.1038/ncomms12695 (2016).

9. Teschner, D., Plescher, J., Piccinin, S., Jones, T. E., Hammud, A., Schmidt, F., Knop-Gericke, A., Bluhm, H. & Shavorskiy, A. Understanding Anomalous Gas-Phase Peak Shifts in Dip-and-Pull Ambient Pressure XPS Experiments. *Journal of Physical Chemistry C* **128,** 7096–7105; 10.1021/acs.jpcc.4c00113 (2024).
